# SNCA-AS1 in aging and Parkinson’s disease

**DOI:** 10.18632/aging.204025

**Published:** 2022-04-18

**Authors:** Stephana Carelli, Federica Rey, Cristina Cereda

**Affiliations:** 1 Pediatric Research Center “Romeo ed Enrica Invernizzi”, Department of Biomedical and Clinical Sciences "L. Sacco", University of Milano, Milano 20157, Italy; 2 Department of Women, Mothers and Neonatal Care, Children's Hospital "V. Buzzi", Milano 20154, Italy

**Keywords:** SNCA-AS1, alpha-synuclein, SNCA, long non-coding RNA, Parkinson's disease

For several years, epigenetics and in particular the study of RNA molecules has attracted the attention of researchers engaged in the study of complex diseases such as cancer. More recently, this field has become of interest also for those who deal with diseases and conditions underlying neurodegeneration. We have identified a long non-coding RNA that regulates synuclein and through its study we were able to have a new outlook on the cellular processes in which it is involved, specifically cellular aging and the etiopathogenetic mechanisms in which synuclein is involved (synucleinopathies). Alpha-synuclein (α-syn) is a small 14 kDa protein encoded by the SNCA gene. Its pathological implications are clear, as it is the main component of Lewy bodies, critical hallmark of Parkinson’s Disease (PD) and of those neurological diseases defined as synucleinopathies [1]. Less is known about its physiological role, although studies suggest the implication for the protein in synapses and synaptic vesicles release [2,3]. An even less characterized aspect of α-syn biology is the regulation of its expression and gene locus, and to this end an antisense gene to SNCA, SNCA-AS1, has been identified in 2014 with an integrated “omics” study performed in human specific biospecimens [4]. SNCA-AS1 encodes for a long non-coding RNA, a class of molecules longer than 200bp which does not code for protein but it is rather implicated in gene expression regulation [5]. Up to now, very limited is the evidence and information available in literature pertaining SNCA-AS1 role and mechanism of action. Indeed, there is only one study which highlights an increased expression of this lncRNA during differentiation of the SH-SY5Y neuroblastoma cell line and iPSCs differentiated into dopaminergic neurons [5]. Moreover, the SNCA locus, including SNCA-AS1, has been recently associated to hereditary neurological diseases and Lewy body dementia suggesting that both genes could contribute to the disease pathogenesis [6].

**Figure 1:**
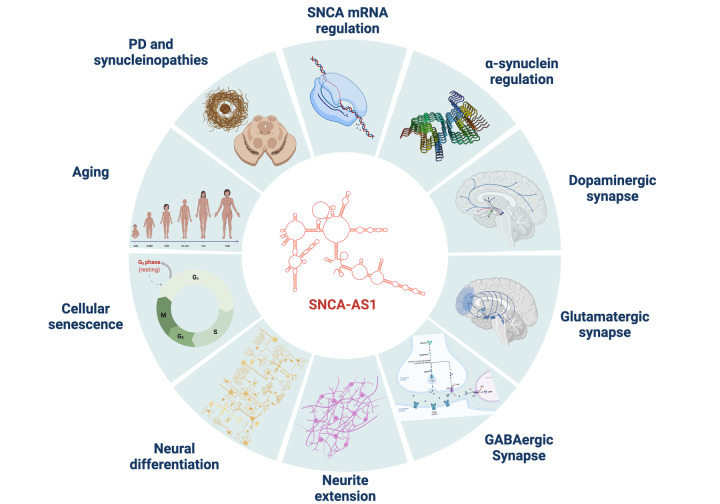
**SNCA-AS1 impact on many cellular processes.** Over-expression of SNCA-AS1 in a neural cell line model (SH-SY5Y) leads to a transcriptional dysregulation correlated with numerous processes. These primarily include SNCA and α-syn regulation, dopaminergic, glutamatergic and GABAergic synapses, neurite extension, neural differentiation, cellular senescence, aging, PD and synucleinopathies. The figure is made with Biorender.com.

In our recent work, we performed a characterization of this lncRNA identifying its transcriptional signature in the in vitro SH-SY5Y cell line [7]. We reported how the overexpression of SNCA-AS1 leads to an increase in SNCA’s mRNA expression, and through RNA sequencing we identified 969 transcripts dysregulated by SNCA-AS1 overexpression and 698 transcripts dysregulated by SNCA overexpression. With both bioinformatic approaches and in vitro validations we described how these genes influence numerous processes, including neurite extension, synaptogenesis, and cellular senescence, all critical processes in both cellular aging and the pathogenesis of synucleinopathies (Figure 1). We also decreased SNCA-AS1 expression using a siRNA targeting its sequence, and we found in this case an opposite effect both on SNCA/α-syn expression and on key targets of dopaminergic pathway [7]. We also believe it is important to emphasize that SNCA-AS1 alone is able to modify these processes and increase α-syn expression, and this can suggest the hypothesis that it in itself could play a role as disease modifying agents or even partially contribute to disease insurgence. Indeed, this lncRNA could be crucial in the regulation of α-syn and PD, with numerous implications also for multiple neurological disorders presenting with synaptic dysfunctions.

Specifically, a novel frontier in neurological disease therapies is represented by “RNA therapy”, a branch of medicine aimed at developing innovative strategies to target genetic and epigenetic aberrations. Indeed, some genetic and epigenetic targets do not respond to traditional drug therapy but on the contrary could be perfect targets for gene/RNA therapy, which is capable of both gene silencing of dominant mutant allele to handle gain of function mutations and gene over-expression, replacing the allele into cells, to handle loss of function mutations [8]. Using viral and non-viral vectors, transgenes that express therapeutic proteins, antibodies, guide RNA (gene editing), microRNAs and small interfering RNA (siRNA) can be delivered to diseased tissues in human and animals [8]. Indeed, a strong RNA dysregulation is present in PD-affected tissues, both in canonical disease-related genes and in novel-genes never before related to the pathology. These can become targets of therapy with precision medicine approaches aimed at inhibiting their expression and the subsequent pathways correction. To this end, several approaches can be used starting from small molecules that target RNA, to more and more specific and refined techniques such as antisense oligonucleotides (ASOs) that bind to a specific homologous sequence or CRISPR/Cas9 System that allows for genome editing [8]. A large number of preclinical studies is present considering these aspects, but advances are also being made in translating these aspects to clinical practice (clinicaltrials.gov). In future prospect, we are convinced that it could thus be possible to develop therapeutic strategies targeting SNCA-AS1, which could prove fundamental in the treatment of synucleinopathies.
